# The Relationship between Single Task, Dual Task Performance and Impulsiveness Personality Traits of Young Adults in the Community

**DOI:** 10.3390/healthcare8040470

**Published:** 2020-11-09

**Authors:** Myoung-Ok Park

**Affiliations:** Department of Occupational Therapy, Division of Health Science, Baekseok University, Cheonan 31065, Korea; parkmo@bu.ac.kr; Tel.: +82-42-550-2185

**Keywords:** impulsiveness, single task, dual task

## Abstract

(1) Background: The aim of this study was to investigate the association between single, dual task performance and impulsiveness personality traits of young adults in the community. (2) Methods: As a cross-sectional study, the Korean version of the Barratt Impulsiveness Scale-11-Revised test, which is an impulsiveness indicator test tool, was conducted on a total of 62 healthy young adults in the community. In order to assess the task performance ability, single task, motor dual task, and cognitive-motor dual task of Timed up and go (TUG) test were conducted. (3) Results: In order to identify the mean difference of the three types of TUG task performance according to the total score of impulsiveness test of all subjects, one-way ANOVA analysis was performed. As a result, there was no statistically significant difference by each task type. Upon investigating the correlation between the three subtypes (cognitive impulsiveness, motor impulsiveness, non-planned impulsiveness) of impulsiveness and total score and TUG task performance, cognitive impulsiveness and TUG-cognitive task performance showed statistically significant correlation. (4) Conclusions: There was an association with the degree of cognitive impulsiveness when performing motor task and cognitive task simultaneously.

## 1. Introduction

The daily tasks performed by humans are not performed as a single task, but have the characteristics of simultaneously applying motor, cognitive and sensory functions in various environments [1]. For example, we can have a conversation while drinking tea, talk while walking, and use mobile phone while driving. As such, human activities are composed of dual-tasks or multi-tasks. Dual task refers to simultaneously performing the target task with one additional task [2]. The patients with neurological cognitive impairment have difficulty performing dual tasks. Because the interference effect in the dual task showed a decrease in simultaneous task performance ability [2]. In information processing, maintaining concentration is an important factor in simultaneously performing two tasks [3]. The ability to process two tasks simultaneously requires proper maintenance of attention capacity and attention allocation for maintaining concentration [4]. Therefore, high performance ability for dual task can be seen as excellent quality of task performance with flexible control of attention maintenance and attention allocation in cognitive information processing process [5]. Based on such evidence, there have been various reports of effects of concentration change and function enhancement in dual task training for patients with stroke and nervous system injury, and children with attention deficit hyperactivity disorder [6,7,8]. However, as mentioned previously, task performance not only involves a single factor, but is also affected by physical and emotional factors, characteristics of the task, and environmental factors in addition to cognitive ability, such as attention [9]. In patients with neurological problems, particularly those who are undergoing occupational therapy, may exhibit not only cognitive decline, but behavioral and emotional changes as well.

Impulsiveness is one of the emotional-behavioral indices, which is generally explained by tendencies and attitudes that show impatient and immediate behavior or lack of long-term, rational thinking [10]. This is used as a basis for understanding many human behaviors and emotions. Impulsiveness includes functional aspects such as rapid decision-making, activity and liveliness, but often, it is considered a personality trait that contributes to dysfunctional and maladaptive behaviors [11]. Extreme impulsiveness and failure of impulse control are known to be closely related to a variety of clinical disorders such as attention deficit hyperactivity disorder (ADHD), borderline personality disorder, antisocial personality disorder, and bipolar disorder [12].

Studies on the effect of impulsiveness on task performance have been mainly conducted in psychological research, but a study investigating the correlation between impulsiveness and behavioral problems in ADHD children showed relevance between the results of Barratt impulsiveness test and reaction time and attention [13].

The present study is aimed at exploring the relationship between impulsiveness personal traits and single and dual task performance in healthy young adults.

## 2. Materials and Methods

### 2.1. Participants

This study was a laboratory based experimental study. The study has been performed at a university laboratory in Cheonan of South Korea from April to May 2017. The participants were recruited from the local university and community group in Chenan. Study enrollment was announced for a total of 8 days from 1 April to 8 April in 2017, and a total of 62 subjects who consented were selected to participate in the study. A research coordinator explained all study procedures to the participants. The information about research ethics were provided to the participants. Written informed consent was gathered form all of participants.

The demographic characteristics of study participants are presented in Table 1. The same ratio of 31 males (50%) and 31 females (50%) participated. All participants did not have any disabilities or orthopaedic/neurological diseases. The age distribution ranged from 18 to 24 years old with the mean age of about 21.34 years old (Table 1).

### 2.2. Measures

#### 2.2.1. Timed Up and Go Test (TUG)

TUG is a test that can quickly assess the basic motility and balance, where the amount of time to sit in a chair with armrests, walking a distance of 3m and returning to the seated position in the chair [14]. TUG test is composed of 3 types of task performances. TUG-alone task is a single task performance, where the subject has to walk a distance of 3m and return to the chair to sit. TUG- motor dual task is composed of a dual task, where the subject has to maintain a cup filled with water in one hand while walking the 3 m distance. TUG-cognitive dual tasks consist of cognitive tasks and motor tasks. When the subject begins walking a distance of 3 m, the examiner selects one of 20 or more digits, presents it to the subject, and is asked to count backwards from the selected number to zero.

In a previous study, TUG test is used as a tool to predict balance and fall [15]. In this study, it was used as a performance scale for single task, motor- dual task, and cognitive-motor dual task.

#### 2.2.2. Korean Version of the Barratt Impulsiveness Scale-11-Revised (K-BIS-11-R)

As for the impulsiveness test, K-BIS-11-R was used. This test is a self-reporting test developed by Patton et al. [16] to assess impulsiveness, consisting of 3 subcategories of 8 questions for cognitive impulsiveness, 11 questions for motor impulsiveness, and 11 questions for non-planned impulsiveness. The rating score is based on how well each question explains oneself, in a 4 point scale ranging from 1 (never) to 4 (always). Cognitive impulsiveness questions assess the tendency of responding without thinking deeply or not carefully making the decisions for problem solving. Motor impulsiveness assesses the tendency of uncontrollable, spontaneous behavior, and non-planned impulsiveness assesses the tendency of not planning or considering stability [17]. Higher total score indicates higher impulsiveness.

### 2.3. Study Process

The experiment was conducted in a pre-set experimental space for all subjects who consent to participate in the study. First, the experimental environment was set up by measuring the distance of 3 m in length and 50 cm in width, and a chair was set to allow a subject to sit. Subjects performed TUG-alone task, TUG-motor dual task and TUG-cognitive motor dual tasks without leaving the 3 m distance as soon as ‘Start’ signal was given by the evaluator.

**Figure 1:**
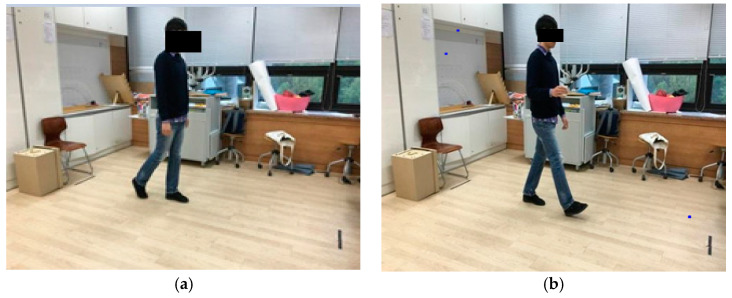
Examples of Dual tasks using Timed up and Go test (TUG). (**a**) TUG-motor dual task, the subject walks with a glass of water in his hand; (**b**) TUG-Cognitive motor dual task, the subject walks while counting backwards.

For TUG-alone task, subjects were to simply walk away and come back. For TUG-motor dual task, subjects had to maintain a cup full of water while walking away and coming back. For TUG-cognitive motor dual task, the evaluator selected a variety of numbers 20 or greater, and the subjects had to walk while counting the given number backwards. The evaluator measured the time it took to perform each task. Impulsiveness test was conducted in a questionnaire format after completing the three tasks (Figure 1).

### 2.4. Analysis Method

For statistical analysis, SPSS 20.0 version ((IBM Corp., Armonk, NY, USA) was used, and the overall statistical significance was set to 5% or less. Descriptive statistics was used for the general characteristics of the study subjects. For the difference of the three TUG tasks among impulsiveness levels, one-way ANOVA was performed. The impulsiveness level was analyzed by classifying the BIS score of the participants in this study as a dummy variable. The dummy criterion was presented by rating the distribution within the group participating in this study by referring to the research results of Lee and colleagues [18].

In this study, 3 distributions were analyzed by dummy processing. Range of total score of Barratt Impulsiveness Scale, (1) is 45–65; (2) is 66–75; (3) is 76–91. Differences within the group were tested by Scheff’s post-hoc test.

Furthermore, for the correlation between the type of impulsiveness and 3 types of TUG task performance, Pearson’s co-efficient was used.

## 3. Results

### 3.1. Impulsiveness Total Score of All Subjects and Difference in Performance of Three Types of Task

In order to identity the mean difference in the three types of TUG task performance and impulsiveness, one way ANOVA was performed.

As a result, there was significant differences between the TUG-cognitive motor dual task among impulsiveness traits (*p* = 0.045). However, as a result of the Scheffe post-hoc test, there was no difference on within group (Table 2).

### 3.2. Correlation of Three Task Performance by Impulsiveness Type of All Subjects

There was a statistically significant correlation between the cognitive impulsiveness type and TUG-cognitive motor task performance when determining the correlation between three types of TUG tasks performed and total score of impulsiveness test of all subjects (r = 0.289 *p* = 0.023). However, there were no significant relations between the other outcomes (*p* > 0.05) (Table 3).

## 4. Discussion

The purpose of this study was to determine the difference in three types of task performance based on impulsiveness and Timed Up and Go test (TUG) in nonclinical adults. According to the study results, there was no difference between the single motor task, and dual motor task group among the level of impulsiveness score. However, there were significant difference in the TUG-cognitive motor dual task by the level of impulsiveness in the subjects. These results can be estimated that the cognitive motor processing speed varies according to the degree of impulsiveness level. The previous study has also mentioned this issue. Research by Shun et al. reported that heavy multitasker exhibited an impulsive and careless aspects, and that it suppressed motor response better than cognitive responses [19]. Activities in everyday life consist mostly of activities that must be handled simultaneously for various stimuli [20]. To do so, the suppression and control of cognitive information processing must be refined [21]. If the impulse tendency is strong, it can be assumed that the cognitive inhibitory ability has reduced the ability to process information at the same time for various stimuli. Previous studies also suggest such a point. In the study of Helton [22], the control of impulse was investigated by presenting a task to maintain sustained attention in order to find out the response of task performance in normal adults. As a result, it was reported that the simultaneous auditory-visual stimulation provided for continuous task performance was useful for controlling impulse. That suggests that performing a dual task with a variety of cognitive information can be handled in a more refined manner only with more impulsive control than with a single task that is automatically performed.

When determining the correlation between the total score for impulsiveness and the three types of TUG task performance, statistically significant correlation was found between cognitive impulsiveness and TUG-cognitive motor dual task performance. According to previous studies, people with impulsiveness tend to perform complex tasks with increased difficulty relatively more accurately than easier tasks [23]. This can be explained as a strategy of utilizing the inhibition function that controls impulsiveness. In the study by Lee [24], generally an impulsive person does not always respond quickly to the cognitive tasks when performing cognitive task performance with high level of difficulty. In this study, it was inferred that the ability to control cognitive impulsiveness has to be good for performing motor and cognitive tasks simultaneously to execute dual tasks requiring motor and cognitive tasks more smoothly. According to Braddeley et al. [25], central executive is deeply associated with working memory and that this is one of the abilities that process two tasks simultaneously. Patients with frontal lobe injury were found to have difficulties in controlling impulsiveness and performing dual asks. Furthermore, a study of children with ADHD tendencies reported issues in the method of performing dual tasks unlike nonclinical children [26]. This finding is evidence that supports the results of this study reporting that the cognitive dual task performance and impulsiveness have higher association than that of the motor dual task performance and impulsiveness indicators. Research by Fischer and colleagues argued that cognitive control ability is a major factor in determining the quality of dual task performance [21]. Cognitive flexibility is related to attention control [27]. It is necessary to identify the factors of priority that affect the quality of task performance by examining the relationship and difference between attention control and impulsive tendency that appear when performing dual task and single task. According to the results of this study, it was confirmed that impulsive subjects tended to process less cognitive-motor dual tasks. However, when dealing with the cognitive-motor dual task, it is necessary to determine whether the participant has prioritized the information processing among the cognitive task and the motor task. Therefore, in future studies, research is needed to understand what priority strategies the subjects have adopted according to their level of impulse. In this study, dual task performance in a small number of young nonclinical adults, but future research needs to examine the relationship between cognitive impulsiveness and task performance skills of the elderly living in the community and develop intervention techniques to solve them.

## 5. Conclusions

The purpose of this study was to identify the difference in impulsiveness, and single task and dual task performance in young nonclinical adults. According to the study results, there was no significant correlation between impulsiveness indicators and single task or motor tasks, but significant correlation was observed in the case of cognitive impulsiveness with single task, motor dual task and cognitive-motor dual task. Such findings suggest that there is difference in the tendency of impulsiveness and quality of task performance.

## Figures and Tables

**Table 1:** General characteristics of all participants (*N* = 62).

Variable	Frequency (%)	Mean ± SD (Range)
Sex		
Male	31 (50)	
Female	31 (50)	
Age		21.34 ± 1.51 (18–24)
Specific illness		
Yes	0 (0)	
No	62 (100)	
Education Level		
Below high school	0 (0)	
Above college	62 (100)	

**Table 2:** Mean difference of 3 types TUG tasks by impulsiveness total score.

TUG	Mean ± SD (s)	F	*p*-Value	Scheffe
TUG-alone task (a)	(1) 8.62 ± 1.36			
	(2) 8.51 ± 1.27	0.154	0.875	(a) = (b) = (c)
	(3) 8.86 ± 1.29			
TUG-motor dual task (b)	(1) 9.21 ± 1.42			
	(2) 9.15 ± 1.28	0.110	0.896	(a) = (b) = (c)
	(3) 9.46 ± 1.36			
TUG-cognitive motor dual task (c)	(1) 8.51 ± 1.84			
	(2) 9.69 ± 2.16	3.272	0.045 *	(a) = (b) = (c)
	(3) 8.99 ± 1.94			

* *p* < 0.05.; Range of total score of Barratt Impulsiveness Scale, (1) is 45–65; (2) is 66–75; (3) is 76–91.; TUG = Timed Up and Go Test.

**Table 3:** Correlation of three types of TUG task performance according to each impulsiveness type.

BIS	TUG-Alone Task		TUG-Motor Dual Task		TUG-Cognitive Motor Dual Task	
	r	*p*-Value	r	*p*-Value	r	*p*-Value
BIS-total	0.012	0.925	0.033	0.800	0.235	0.065
BIS-Motor	−0.057	0.659	−0.072	0.578	0.158	0.220
BIS-Cognitive	0.119	0.359	0.081	0.531	0.289	0.023 *
BIS-non plan	0.015	0.905	0.065	0.618	0.153	0.234

* *p* < 0.05; r = Pearson’s correlation co-efficients; BIS = Barratt Impulsiveness Scale; TUG = Timed Up and Go Test.
